# Plateau Pika Disturbance Changes Soil Bacterial Functions and Exoenzyme Abundance to Modulate the C Cycle Pathway in Alpine Grasslands

**DOI:** 10.3390/ijms252312775

**Published:** 2024-11-28

**Authors:** Jing Li, Qing Wang, Baolong Zhu, Min Yang

**Affiliations:** 1 School of Civil Engineering and Architecture, Southwest Science and Technology University, Mianyang 621010, China; jli@swust.edu.cn (J.L.); miro-y@swust.edu.cn (M.Y.); 2 School of Environment and Resource, Southwest Science and Technology University, Mianyang 621010, China; qingw@imde.ac.cn

**Keywords:** *Ochotona curzoniae*, Qinghai–Tibetan plateau, soil organic carbon, soil microorganisms

## Abstract

Plateau pika (*Ochotona curzoniae*) is crucial to soil organic carbon (SOC) storage in the Qinghai–Tibetan plateau (QTP), but its role in bacterial SOC metabolisms across different degraded alpine grasslands remains unclear. In this study, we investigated the soil physicochemical properties and the composition and function of the bacterial communities in control and pika-disturbed grasslands experiencing different degradation levels (undegraded, UDM; lightly, LDM; moderately, MDM and severely, SDM). The results demonstrate that (i) the primary bacterial phyla include *Proteobacteria*, *Acidobacteriota*, *Actinobacteriota*, *Bacteroidota* and *Verrucomicrobiota*. Soil physicochemical properties significantly impact the composition of the bacterial communities and determine the influence of pika disturbance. Pika disturbance increases bacterial OTUs by 7.5% in LDP (*p* > 0.05) and by 50.5% in MDP (*p* < 0.05), while decreases OTUs by 21.4% in SDP (*p* < 0.05). (ii) Pika disturbance downregulates the exoenzyme abundance associated with simple and complex organic matter decomposition by 9.5% and 13.9% in LDP, and 29.4% and 26.3% in MDP (*p* < 0.05), while upregulates these exoenzymes by 23.6% and 37.9% in SDP (*p* < 0.05). These changes correspond to the increase in TC and SOC in LDP and MDP but declines in SDP. (iii) Plateau pika disturbance can enhance SOC accumulation through upregulating the C cycle pathway of ethanol production in LDP and MDP. However, it upregulates the pathway of pyruvate to CO_2_ conversion in SDP, leading to negative influence on SOC storage.

## 1. Introduction

The alpine grasslands in the Qinghai–Tibetan plateau (QTP) are estimated to store 23.2 Pg of soil organic carbon (SOC), making them vulnerable to environmental disturbance [1,2,3]. Given the significant SOC storage, even minor alterations in SOC metabolisms within alpine grasslands will exert a profound impact on the global carbon (C) cycle [4,5,6]. To date, numerous environmental factors have been proposed as regulators of SOC metabolisms in alpine grasslands, including climate change, human activities, topography variations, heterogeneity in soil properties and animal disturbance [5,7,8,9,10,11,12,13,14,15].

Plateau pika (*Ochotona curzoniae*) is a native small mammal in the QTP [16,17], widely distributed in alpine grasslands, and its density is expected to increase with climate warming in the QTP [18,19]. As the key species of the alpine biomes in the QTP, previous studies have examined the impact of plateau pika disturbance on SOC storage [17,20,21,22], but mostly focused on its effect on plant C input and/or bare patch formation [23,24,25]. It has been recognized that the pika’s phytophagous habits could damage SOC storage due to vegetation consumption [26,27,28]; however, this hypothesis hardly explained the observed increase in vegetation diversity and SOC content in pika-disturbed alpine grasslands [23,24,29]. On the other hand, the plateau pika’s burrowing activities were another reason contributing to SOC storage deterioration [25,30,31,32], as they caused bare patch formation, especially at their burrowing entrances, which significantly reduced alpine grassland stability and promoted SOC loss [30,31,33,34,35]. However, the connected underground tunnels of plateau pika can also promote fluid infiltration and nutrient circulation, which benefit plant growth and SOC accumulation [17,20,24]. Therefore, more factors need to be investigated to reveal the role of plateau pika in SOC circulation.

The microbial catalysis and decomposition of SOC serve as the fundamental mechanisms controlling both SOC storage and the C cycle in alpine grasslands [13,14,15]. Previous investigations have indicated that plateau pika disturbance can directly influence soil microbial communities by altering their composition [36], and/or indirectly impact them by modifying their microenvironment, such as soil moisture, pH, bulk density, nutrient and aeration [34,37,38]. However, the role of plateau pika involving SOC metabolisms and C cycle pathways in alpine grasslands remains largely unknown. Furthermore, significant variations in soil physicochemical properties occur in alpine grassland with different degrees of degradation, resulting in heterogeneity in the microenvironment of microbial communities [37,39,40]. It is also unclear whether and how these differences affect the role of plateau pika in SOC metabolisms.

Therefore, this study investigates the soil physicochemical properties, SOC content, composition and functions of the bacterial communities, and exoenzyme activities in control and pika-disturbed grasslands experiencing different degradation levels (undegraded, UDM; lightly, LDM; moderately, MDM and severely, SDM). Our primary target is to reveal the impact of plateau pika on bacterial communities associated with SOC metabolism and C cycle pathways, as well as export their variations across different degraded grasslands in the QTP.

## 2. Results

### 2.1. Plateau Pika Disturbance Changes Soil Physicochemical Properties

According to WRB [41], the soil in the sampling area can be classified as Kastanozems, with a thickness of humus horizon ranging from 10 cm to 40 cm, which decreases as the degradation level increases. The variations in soil pH, water content (SWC), bulk density (SBD), soil texture (sand, silt and clay), soil nitrogen (TN) and its assimilable forms (ammonium and nitrate nitrogen (NH^4+^-N and NO^3−^-N)), total C (TC), SOC and microbial biomass carbon (MBC) across different alpine grasslands are shown in Table 1.

Soil pH significantly varies across different grasslands and is between 5.39, 5.72, 4.81 and 7.11 in UDM, LDC, MDC and SDC (control sites of LDM, MDM and SDM), while pika disturbance has a negligible impact on soil pH (*p* > 0.05). SWC, clay, silt, TC, TN and SOC significantly decrease with an increasing degradation level, while sand and C:N exhibit an opposite pattern. Plateau pika disturbance increases SWC by 6.6% and 18.5%, TC by 5.1% and 10.1%, and SOC by 4.4% and 10.5% in LDP and MDP (pika-disturbed sites of LDM and MDM), respectively. But it decreases SWC by 12.2%, TC by 11.5%, and SOC by 13.1% in SDP (pika-disturbed sites of SDM) (*p* < 0.05). Meanwhile, for TN, pika disturbance significantly decreases it by 17.4%, 10.3% and 11.6% in LDP, MDP and SDP (*p* < 0.05); therefore, the C:N decreases in all grassland. Pika disturbance increases the ratio of sand while decreasing the ratio of clay and silt, but these changes are not significant (*p* > 0.05). MBC is relatively high in UDM and MDC, while relatively low in LDC and SDC; pika disturbance significantly increases it by 29.6%, 54.5% and 25.8% in LDP, MDP and SDP, respectively (*p* < 0.05). SBD is not closely associated with the degradation levels, with the rank ranging from high to low as follows: LDC > SDC > MDC > UDM. Plateau pika disturbance reduces SBD in all types of alpine grasslands and is particularly pronounced in LDP (*p* < 0.01). The NH^4+^-N rank ranging from high to low is as follows: UDM > SDC > MDC > LDC. Meanwhile, for NO^3−^-N, it is as follows: UDM > LDC > MDC > SDC. Pika disturbance increases both NH^4+^-N and NO^3−^-N in all grassland (*p* < 0.05), although it slightly decreases NH^4+^-N in SDP (*p* > 0.05).

### 2.2. Plateau Pika Disturbance Alters Bacterial Communities’ Functions and Exoenzyme Abundance

#### 2.2.1. Bacterial Communities’ Abundance and Diversity

The abundance and diversity of bacterial communities vary significantly across different alpine grasslands. As depicted in Table 2, the observed operational taxonomic units (OTUs) follow the following rank from low to high: MDC < LDC < UDM < SDC. Plateau pika disturbance increases OTUs by 7.5% and 50.5% in LDP (*p* > 0.05) and MDP (*p* < 0.05), while it decreases OTU by 21.4% in SDP (*p* < 0.05).

The *α* diversity is positively correlated with degradation levels, despite it being relatively high in UDM. Pika disturbance increases Chao_1 and Faith_pd in LDP and MDP, while it decreases them in SDP. For the Shannon_Winner index, it follows the rank from low to high of UDM < LDC < SDC < MDC; pika disturbance decreases it in LDP and SDP, while it slightly increases it in MDP. There is no obvious connection between *β* diversity and soil properties or degradation levels of alpine grasslands. Plateau pika disturbance only significantly increase Bray_curtis and Weighted Unifrac in SDP. However, these changes are not statistically significant (*p* > 0.05).

#### 2.2.2. Bacterial Communities’ Composition

**Figure 1:**
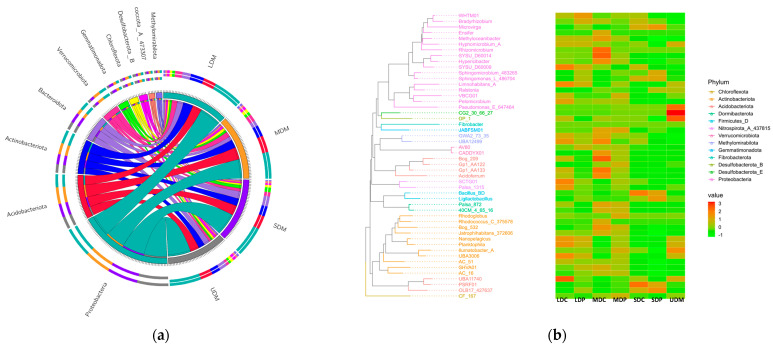
Bacterial specificity in control and plateau pika-disturbed alpine grasslands. (**a**) Bacterial phyla abundance; (**b**) Heatmap cluster analysis for the composition of bacterial communities.

Figure 1a shows that the primary bacterial phyla include *Proteobacteria*, *Acidobacteriota*, *Actinobacteriota*, *Bacteroidota* and *Verrucomicrobiota*. The heatmap cluster analysis in Figure 1b reveals significant differences across different alpine grasslands. In UDM, the particularly enriched phyla included *Desulfobacterota* and *Nitrospirota*, whose abundance is also relatively high in LDC. In MDC, the phyla of *Methylomirabilota* and *Acidobacteriota* are relatively enriched. Meanwhile in SDC, the phyla of *Gemmatimonadota* and *Planctomycetota* are relatively dominant.

Plateau pika disturbance decreases the original dominant bacterial communities while simultaneously upregulating other bacterial communities. These effects varied across different alpine grasslands. In LDP, plateau pika disturbance improved the relative abundance of *Proteobacteria*, *Actinobacteriota*, *Verrucomicrobiota* and *Firmicutes*. In MDP, pika disturbance upregulated the relative abundance of *Eisenbacteria* and *Chloroflexota*. In SDP, the original dominance state of *Gemmatimonadota* and *Planctomycetota* remained, and pika disturbance further upregulated the phyla of *Firmicutes*, *Bacteroidota*, *Myxococota* and *Proteobacteria*.

**Figure 2:**
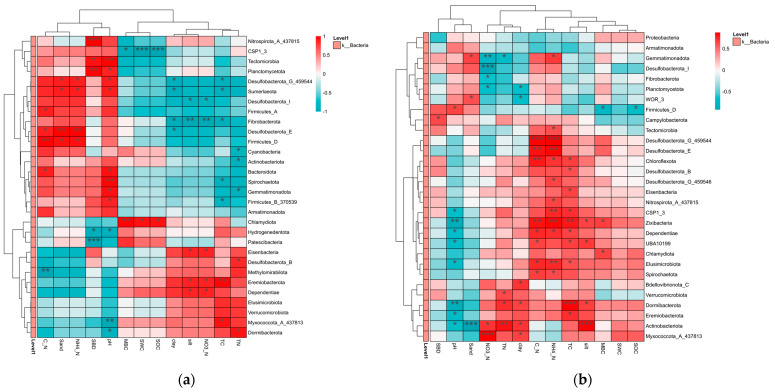
Correlations between soil physicochemical properties and the composition of bacterial communities in control (**a**) and plateau pika-disturbed (**b**) alpine grasslands. * *p* < 0.05, ** *p* < 0.01, *** *p* < 0.001.

Soil physicochemical properties significantly impact bacterial community composition across various alpine grasslands. As depicted in Figure 2a, soil pH, SBD, C:N, sand and NH^4+^-N are primarily positively correlated to the phyla of *Desulfobacterota*, *Firmicutes*, *Gemmatimonadota* and *Planctomycetota*, while other properties are mostly negatively correlated to these phyla and are positively correlated to *Desulfobacterota* and *Firmicutes.*

Plateau pika disturbance enhances the positive effect of SBD, C:N, sand and NH^4+^-N silt, clay, TN and TC in the phyla *Desulfobacterota*, *Zixibacteria*, *Domibacterota*, *Myxococcota*, *Actinobacteriota*, *Eremiobacterota* and *Dependentiae*, while it decrease the positive effect of soil pH, SBD and sand on microbial composition (Figure 2b).

**Figure 3:**
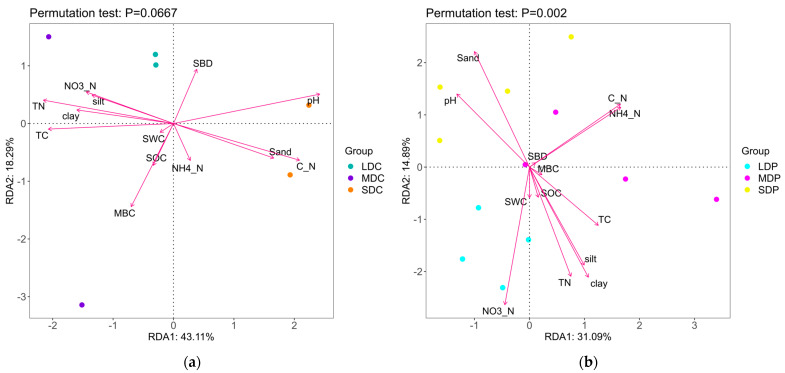
Redundancy analysis for the effect of soil physicochemical properties on the composition of bacterial communities in control (**a**) and pika-disturbed (**b**) alpine grasslands.

RDA analysis provides further evidence of the differences in correlations between control and plateau pika-disturbed alpine grasslands (Figure 3). In control grasslands (Figure 3a), soil pH, sand, C:N, SBD and NH^4+^-N are the positive factors correlated with bacterial composition, while TC, TN, clay, silt and NO^3−^-N are the primary negative factors. However, pika disturbance conversely affects the impact of sand, soil pH, TN, TC, silt and clay on bacterial composition, enhancing the positive effect of NH^4+^-N while reducing the influence of SBD, MBC and NO^3−^-N in regulating bacterial communities (Figure 3b).

#### 2.2.3. Bacterial Communities Function

**Figure 4:**
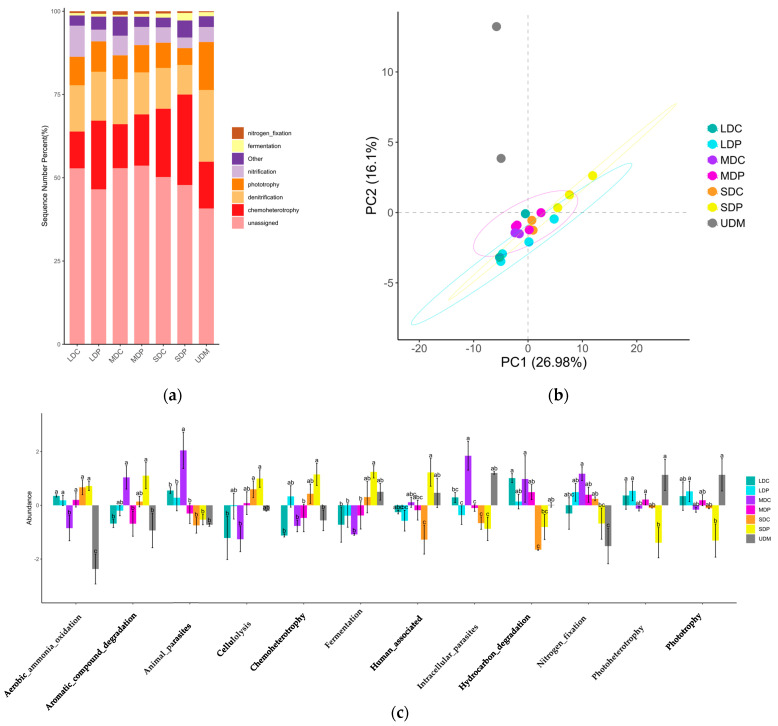
Bacterial communities’ functions in control and plateau pika-disturbed alpine grasslands. (**a**) Grouped percentage stacked bar chart for bacterial functions; (**b**) Principal component analysis for the bacterial functions; (**c**) ANOVA + Duncan grouped comparison bar chart for bacterial functions. The letters refer to the significant differences among varied degraded alpine grasslands (*p* < 0.05).

To discern the microbial function, the predicted 16S genes were compared to the Functional Annotation of Prokaryotic Taxa (FAPROTAX) database, and the results are shown in Figure 4.

The bacterial functions primarily encompass chemoheterotrophy, prototrophy, nitrification and denitrification (Figure 4a). The PCA results demonstrate significant dissimilarities in the bacterial function genes, particularly in UDM. Plateau pika disturbance further amplifies these disparities across different alpine grasslands, with a particularly pronounced effect observed in SDP (Figure 4b). In particular, genes related to prototrophy and photoheterotrophy are more abundant, while genes related to chemotrophy, especially cellulose degradation, are observed to be less abundant in UDM. This suggests that the carbon and energy source may rely more on photosynthesis by utilizing inorganic carbon. Conversely, an opposite pattern is found in SDC, where genes related to the decomposition of complex organic matter, such as cellulose and aromatic compounds, are more abundant, while genes related to photoautotrophy and photoheterotrophy are less abundant, suggesting that soil microorganisms tend to rely more on decomposing organic matter for growth and reproduction. In MDC, genes associated with nitrogen fixation, hydrocarbon degradation and aerobic ammonia oxidation are more prevalent compared to others. Plateau pika disturbance has differential impacts on bacterial function genes across different alpine grasslands. For instance, plateau pika disturbance increases the abundance of photoheterotrophy and photoautotrophy in LDP and MDP but decreases them in SDP. In MDP, plateau pika disturbance leads to a decline in chemotrophy, aerobic ammonia oxidation and hydrocarbon degradation gene abundance. However, in SDP, plateau pika disturbance upregulates the abundance of genes related to aromatic compound degradation (Figure 4c).

#### 2.2.4. Exoenzyme Abundance

**Figure 5:**
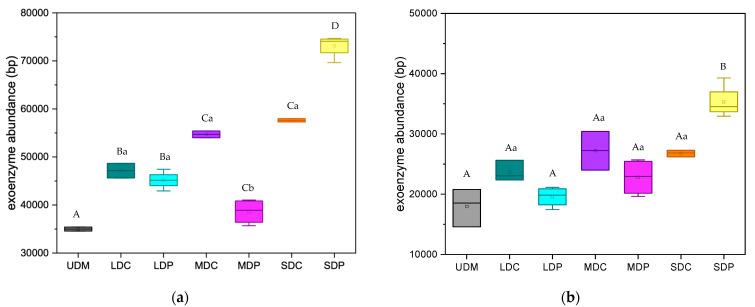
Exoenzyme for relatively simple (**a**) and complex (**b**) organic matter decomposition in control and plateau pika-disturbed alpine grasslands. The uppercase letters indicate the significant difference among varied degraded alpine grasslands; the lowercase letters refer to the significant differences between control and plateau pika disturbed alpine grasslands (*p* < 0.05).

The exoenzyme abundance associated with organic matter decomposition was analyzed by comparing the Kyoto Encyclopedia of Genes and Genomes (KEGG) http://www.kegg.jp/ (accessed 11 May 2024) database, and the results are shown in Figure 5. Both the abundance of the exoenzyme associated with relatively simple organic matter decomposition (starch, hemicellulose, cellulose and pectin) (Figure 5a) and complex organic matter decomposition (chitin and aromatics) (Figure 5b) are positively correlated with the degradation level. Plateau pika disturbance led to decreased exoenzyme abundance in LDP and MDP but increased that in SDP, resulting in significant variations in exoenzyme abundance across pika-disturbed grasslands.

### 2.3. Plateau Pika Disturbance Modulates C Cycle Pathways in Alpine Grasslands

**Figure 6:**
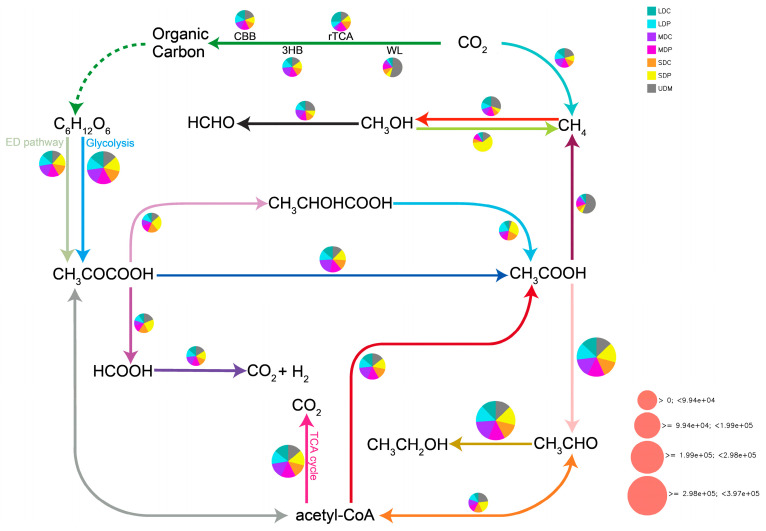
C cycle pathways across different alpine grasslands. CBB: Calvin–Benson–Bassham cycle; 3HB: 3-hydroxybutyrate pathway; rTCA: reductive tricarboxylic acid pathway; WL: Wood–Ljungdahl pathway; acetyl-CoA: acetyl-CoA; TCA cycle: tricarboxylic acid cycle. The size of the pie chart represents the relative abundance of the metabolic pathway. Arrows indicate chemical reaction pathways. The direction of the arrows is from substrate to product in the chemical reaction. Different colors represent different pathways. Bidirectional arrows indicate reversible chemical reaction pathways. Dashed arrows indicate that there is no relevant gene (KO) information, hence there is no abundance for this pathway, and no pie chart is drawn. Arrows without pie charts represent that no relevant genes (KO) for this pathway were detected in the sample.

Based on the abundance of KEGG Orthology, the C cycle pathways were calculated by DiTing software (version 0.9) http://github.com (access on 1 May 2024), and the results are shown in Figure 6. The primary pathways in translating CO_2_ into organic carbon include the Calvin–Benson–Bassham cycle (CBB), 3-hydroxybutyrate pathway (3HB), reduced TCA cycle (rTCA) and Wood–Ljungdahl pathway (WL). Consistent with the predominant bacterial phyla of *Desulfobacterota* and *Nitrospirota*, the WL pathway is particularly predominant in UDM due to its strict occurrence under aneoretic conditions. This condition in UDM may also result in a significant proportion being involved in the reduction in acetic acid (CH_3_COOH) to methane (CH_4_), which algins with previous investigations [42]. Notably, the reduction in methanol (CH_3_OH) to CH_4_ is particularly pronounced in SDP, and these suggest that pika disturbance in SDP may lead to a more significant impact on global climate change as CH_4_ is a more potent greenhouse gas than CO_2_ [42].

## 3. Discussion

### 3.1. The Composition of Bacterial Communities Is Primarily Determined by Soil Physicochemial Properties

In this study, we observe that bacterial community composition varies significantly across alpine grasslands experiencing different degradation levels, and these variations can be primarily attributed to the varying background soil physicochemical properties. For instance, the extremely high SWC in UDM creates an anaerobic environment that favors the growth of *Deltaproteobacteria* and *Nitrospirae* [42,43]. These bacterial phyla facilitate anaerobic sulfate and anoxic nitrate reduction reactions, which inhibit organic matter decomposition and enhance SOC accumulation. On the other hand, the high percentage of fine particles, including clay and silt, also prohibit the transfer of oxygen; therefore, a relatively high abundance of *Deltaproteobacteria* and *Nitrospirae* also persists in LDC due to the high SWC and SBD as well as abundant clay and silt, contributing to the formation of an anaerobic environment. In MDC, the lowest soil pH result in an acidic oxidative environment favors the proliferation of *Methylophilaceae* and *Acidobacteria* [8,44]. Meanwhile, in SDC, the low SWC and SOC content create an oligotrophic environment, where the phyla of *Gemmatimonadetes* and *Planctomycetes* are relatively abundant. This is because most species within these phyla thrive in harsh environments and adapt to oligotrophic strategies [45]. Moreover, the highest content of sand in SDC can improve soil aeration, thereby enhancing microorganism growth and reproduction, resulting in the highest abundance and diversity observed in SDC.

Plateau pika disturbance can directly alter the native soil composition of the bacterial communities by introducing pika-specific microorganisms (primarily gut microorganisms) [36,46] or indirectly through modifying the microenvironment [36,47,48]. However, these direct and indirect effects are significantly differential across alpine grasslands experiencing different degradation levels, which may be due to the variations in their background soil physicochemical properties. For instance, the relatively low soil pH and high SWC in LDP and MDP can create a microenvironment close to the intestinal environment of plateau pika, which facilitate the growth and reproduction of pika gut microorganisms, leading to an increase in the abundance and diversity of bacterial communities [46]. Conversely, the relatively high soil pH and low SWC in SDP will hinder the thrive of pika gut microorganisms, which may be the reason for the decline of bacterial community abundance and diversity. In terms of indirect effects, pika disturbance significantly reduced SBD in LDP, thereby enhancing soil aeration and decreasing the abundance of *Deltaproteobacteria* and *Nitrospirae*. Pika disturbance also increased SWC, TC, SOC, TN, NH^4+^-N and NO^3−^-N, which contributed to the proliferation of *Proteobacteria*, as species in this phylum typically thrive in carbon- and nutrient-rich environments [49]. Pika disturbance increased SWC, thus facilitating the anaerobic environment formation in MDP, resulting in the improvement of *Gallionellaceae* and *Chloroflexi* [50]. Meanwhile, in SDP, pika disturbance decreased SWC, TC, NO^3−^-N and SOC; this rigid environment promoted oligotrophic strategy phyla, including *Gemmatimonadetes*, *Firmicutes*, *Bacteroidetes*, *Myxococcales*, *Proteobacteria*, and *Planctomycetes* [51].

### 3.2. The Bacterial Function and Exoenzyme Abundance Is Determined by Bacterial Composition, Which Significantly Impact SOC Content

The variations in the composition of the bacterial communities result in diverse bacterial functions across different grasslands, which contribute to the differential SOC accumulation in each grasslands type. In UDM, there is a relatively high abundance of bacterial genes related to phototrophy, while genes related to chemoheterotrophy, particularly cellulose degradation, are less abundant. This suggests that carbon and energy sources may primarily rely more on inorganic carbon utilization through photosynthesis, rather than organic carbon decomposition, which correspond to its highest SOC content. Conversely, in SDC, there is a relatively high abundance of bacterial genes related to organic matter decomposition and a low abundance of genes related to photoautotrophy and photoheterotrophy. This indicates that the bacterial communities in SDC may depend more on organic matter decomposition for growth and reproduction, aligning with its lowest SOC content. The impacts of plateau pika disturbance also align with these trends. As discussed above, the influence of pika disturbance on the composition of the bacterial communities is determined by background soil properties; therefore, the bacterial communities’ functions are also controlled by the background soil properties.

On the other hand, the conversion of plant C inputs into soil organic carbon involves the decomposition of plant components by exoenzyme. The quantity and quality of plant C inputs play a crucial role in determining exoenzyme activities. Previous studies have indicated that the energy and nutrient costs of exoenzyme production are particularly high in bare or deep soils [52] since microorganisms evolve in specific microbial communities to cover a wide range of required reactions in these environments [53], thus occurring in more complex microbial community compositions and exoenzyme. In UDM, LDC and MDC, the relatively high SWC as well as C and N resources enhance vegetation growth, thus leading to an abundant and high quality of plant C input. Therefore, microorganisms preferentially utilize easily accessible energy sources while leaving difficult-to-decompose plant residues as organic carbon precursors for accumulating and entering the SOC pool [52,54,55], which is associated with lower levels of exoenzyme [54]. As a result, in UDM, LDC and MDC, exoenzyme abundance is relatively low. Meanwhile, in SDC, the low SWC, in addition to poor C and N resources, may hinder plant growth, resulting in less plant C input and thus microorganisms need to decompose difficult-to-decompose plant residues to meet their growth and reproduction needs, which reduces the plant SOC precursors into the SOC pool. Therefore, bacterial communities’ functions related to organic matter decomposition and exoenzyme abundance are more robustly expressed in SDC.

Pika disturbance leads to a decrease in exoenzyme abundance in LDP and MDP, indicating enhanced efficiency in plant C conversion and greater SOC accumulation. This explains the positive impact of plateau pika disturbance on SOC stocks in these grasslands. Conversely, plateau pika disturbance increases the abundance of exoenzymes associated with organic matter decomposition in SDP, suggesting a reduced efficiency in plant carbon conversion and lower SOC accumulation, which correspond to the declines in TC and SOC in SDP.

### 3.3. The Effects of Plateau Pika Disturbance on C Cycle Pathways Are Differential Across Didfferent Alpine Grasslands

The carbon sink function of grasslands is primarily achieved by capturing inorganic carbon (CO_2_) and fixing it into SOC storage. Our study reveals that the Calvin–Benson–Bassham cycle (CBB), 3-hydroxybutyrate pathway (3-HB), reductive TCA cycle (rTCA), and Wood–Ljungdahl pathway (WL) are the main pathways for converting CO_2_ into organic carbon. Among these pathways, the WL pathway exhibits a particularly dominant role in UDM due to its anaerobic conditions. In contrast, LDC displays a higher abundance of functional genes related to photoautotrophic and photoheterotrophic pathways, thereby favoring the rTCA pathway. The impact of plateau pika disturbance on carbon cycle pathways primarily lies in its influence on pyruvate conversion to lactic acid, formic acid or acetic acid. Notably, we observe higher abundances in pika-disturbed grasslands compared to control sites. Furthermore, our findings indicate that acetic acid conversion to ethanol is much higher than that to methane, suggesting that most acetic acid can be converted to ethanol and enter the SOC pool via this particular pathway. These effects can partially explain the positive impact of plateau pika disturbance on SOC content. However, the dominant position of plateau pika disturbance in SDP in pathway abundance seems contradictory to the result that plateau pika disturbance leads to a decrease in SOC content in SDP. This discrepancy may be reconciled by the pathway of pyruvate; its conversion to CO_2_ and hydrogen (H_2_) through formic acid also shows the highest abundance in SDP. This indicates that plateau pika disturbance can accelerate CO_2_ emission in these SDP, which is also consistent with previous findings that the CO_2_ content in pika burrow gas was significantly higher than the atmospheric concentration [56].

Another one of our findings is that the pathway abundance of methanol reducing to CH_4_ is significantly higher in SDP, indicating that plateau pika disturbance can enhance CH_4_ formation and release in SDP. Previous studies have pointed out that CH_4_ emissions increase in alpine grasslands under plateau pika disturbance, but that study did not examine the differences in the degradation levels of alpine grasslands [56]. Our study suggests that plateau pika disturbance not only significantly affects CO_2_ emissions but also CH_4_ emissions in different degraded alpine grasslands. Since CH_4_ has a greenhouse effect about 25 times that of CO_2_ [57], the impact of plateau pika disturbance on CH_4_ emissions and its differential effects in different degraded grasslands will be further explored in our future research.

Notably, the present study is focused on the influence of pika disturbance on bacterial functions and exoenzyme abundance, but other organisms such as fungal communities may also impact the SOC microbial metabolisms. Moreover, *Marmota himalayana* is also distributed in these areas, and the pika tunnels are also the habitats of many birds, such as *Montifringilla spp* and *Pseudopodoces humilis*. Whether this wildlife impacted SOC metabolisms and whether there were intersections with plateau pika remains unclear. These issues will be explored in our future research.

## 4. Materials and Methods

### 4.1. Sampling Area and Methods

**Figure 7:**
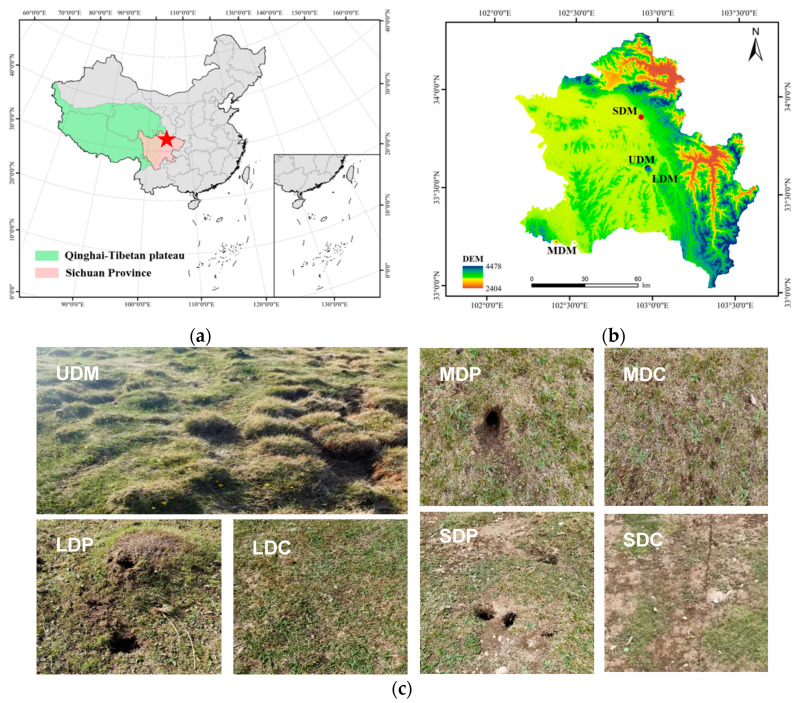
Sampling locations and photos of each sampling site. (**a**) The red pentagram indicates the sampling site; (**b**) Topographic locations for each sampling site. UDM, undegraded alpine grassland; LDM, lightly degraded alpine grassland; MDM, moderately degraded alpine grassland; SDM, severely degraded alpine grassland. (**c**) Photos of each sampling site. LDP and LDC, pika-disturbed and control lightly degraded alpine grassland; MDP and MDC, pika-disturbed and control moderately degraded alpine grassland; SDP and SDC, pika-disturbed and control severely degraded alpine grassland.

Soil sampling was conducted on the eastern edge of the QTP, Tangke town, Zoige county, Sichuan, China. To exclude the influence of altitude, the altitude of all sampling sites were around 3420 m, and the longitude and latitude ranged from 102.412° to 102.957°, and 33.241° to 33.886°, respectively (Figure 7a,b). The climate in this region is a plateau continental climate, with a warm season (June to September) and a cold season (October to May). The mean annual temperature (MAT) is around −1.8 to 1.5 °C, and the lowest and highest temperature occurring in January (−22.5 °C) and August (25 °C), respectively. The mean annual precipitation (MAP) is around 456 mm to 620 mm, and the majority of precipitation takes place from May to August.

Four degradation levels of alpine grasslands were selected for soil sampling, including undegraded (UDM), lightly degraded (LDM), moderately degraded (MDM) and severely degraded (SDM) alpine grasslands. The specific classification is carried out according to previous studies [37,38,40]. As illustrated in Figure 7c, the vegetation coverage percentage in UDM is approximately 90~100%, without wind erosion. The vegetation coverage percentage in LDM is approximately 70~80%, with slight wind erosion. The vegetation coverage percentage in MDM is approximately 50~70%, with slight to moderate wind erosion. The vegetation coverage percent in UDM is less than 50%, with severe wind erosion. In terms of dominant species, Cyperaceae prevails in UDM but gradually transitions into weed species with increasing degradation levels. Specifically, *Stipa purpurea* represents the dominant vegetation species within Cyperaceae, while *Kobresia pygmaea* characterizes the weed vegetation, respectively.

In LDM, MDM and SDM, both control and pika-disturbed soil spots (50 × 50 m) were established, with a separation distance of 50 m. Notably, only the control site was set in UDM, where there was no plateau pika disturbance. For each soil spot, three sample sites (10 × 10 m) were randomly set, soil from the surface layer (0~10 cm) and subsoil layer (20~30 cm) were collected using a soil drill with a diameter of 8.5 cm. Triplicate samples were collected and mixed at the same soil layers to mitigate the influence of soil heterogeneity. For pika-disturbed samples, both the bare soil near pika burrowing entrances and vegetation-covered soil above pika tunnels were collected. After thoroughly mixing the soil sample, it was divided into three parts. One part of the soil was stored at low temperature (4 °C) for the subsequent microbial biomass carbon analysis, another part was stored in dry ice for microbial sequencing, and the remaining part was naturally air-dried for measurements of the soil properties.

### 4.2. Soil Physicochemical Properties Measurement

Soil bulk density (SBD) was measured using the ring knife sampling and drying method. Soil water content (SWC) was measured through the aluminum box drying method. Air-dried soil was diluted with deionized water at a ratio of 1:5 (*w*/*w*), and the pH value was measured using the glass electrode method (SevenExcellence S400, Mettler Toledo, Zurich, Switzerland). Soil sand, silt and clay were measured by a laser particle size analyzer (Mastersizer 3000, Malvern, UK). Air-dried soil was ground and sieved through a 100-mesh sieve, and we measured TC using an automatic carbon analyzer (UIC CM250, Chicago, IL, USA). TN was measured by the Kjeldahl method. The SOC measurement was digested with potassium dichromate (K_2_Cr_2_O_7_), and we determined the SOC content using spectrophotometry [58]. Soil was extract by KCl (2M), then, we measured NH^4+^-N and NO^3−^-N using an autoanalyzer (SEAL, AA3, Mönchengladbach, German). The fumigation extraction method was used for the analysis of microbial biomass C (MBC) [59].

### 4.3. Microbial Sequencing and Metagenomic Funtional Prediction

The CTAB (Cetyltrimethylammonium bromide) method, as described by Nobleryder in China, was used for soil DNA extraction and total genomic DNA amplification. The DNA concentration and quality were assessed using the NanoDrop One spectrophotometer (Thermo Fisher Scientific, Waltham, MA, USA). The taxonomic identification of bacterial taxa was based on the 16S rRNA gene. Specific primers (338F: 5′-CCTAYGGGBGCASCAG-3′) and (806R: 5′-GGACTACNNGGGTATCTAAT-3′) were used to amplify the V3-V4 region of the 16S rRNA gene. All PCR reactions were performed in 15 μL of Phusion^®^. High-fidelity PCR was performed in a main mixture (New England Biolabs, Ipswich, MA, USA) containing 2 μM forward and reverse primers and 10 ng template DNA. The mixture underwent denaturation at 98 °C for 1 min, followed by 30 cycles of denaturation at 98 °C for 10 s, annealing at 50 °C for 30 s and elongation at 72 °C for 30 s. Subsequently, these PCR products were purified using Universal DNA (Tiangen, Beijing, China) to generate a library, and then they were subjected to NEB Next^®^ Ultra DNA. The library preparation kit (Illumina, San Diego, CA, USA) prepared Illumina sequencing libraries according to the manufacturer’s recommendations. The library quality was evaluated on Agilent 5400 (Agilent Technologies Co Ltd., Santa Clara, CA, USA), followed by sequencing on the Illumina platform to generate 250 bp paired-end reads. We used normalized read counts to mitigate the impact of sequencing depth between different groups. We performed chimeric sequence analysis according to the Atacama Soil Microbial Tutorial in Qiime2 document and used custom program scripts https://docs.qiime2.org/2019.1 (access on 15 April 2024) [60]. Simultaneously, we used the dada2 plugin to identify and remove raw data, in order to obtain amplicon sequence variant (ASV) features. Then, we used the QIIME2 feature classifier plugin to align the ASV sequence with the pre-trained Greengenes2 database, generating operational taxonomic units (OTUs) for bacteria. We used the core diversity plugin in QIIME2 to calculate the diversity metrics. Based on the sklearn algorithm for species classification of ASV, the bacterial classification database is the Greengenes2 database (16S).

### 4.4. Statistical Analysis

For the detection of alpha diversity in bacteria and fungi, dilution curves were used to calculate alpha diversity indices, including chao1, Shannon Winner, faith_pd, Simpson index and observed features. The number of sample points was set to 50. The beta diversity was analyzed using nonmetric multidimensional scaling analysis (NMDS) based on the Bray–Curtis distance, weighted Unifrac and unweighted Unifrac. PICRUSt2 software (version 3.0) was applied to predict the metagenome in the sample based on the ASV (or OTU) abundance table and ASV sequence (or representative sequence). The predicted 16S genes were compared with the prokaryotic functional annotation (FAPROTAX) database to annotate bacterial community functions [61]. Functional multiple comparison using one-way ANOVA+Duncan test was applied to search for categories with significant differences across groups and draw a bar chart. The intra group correlation heatmap tests were based on the correlation coefficients between environmental factors. The above analysis was completed using R software (version 4.2.1). Based on the KEGG orthologous abundance, DiTing software was used (http://github.com) to build a carbon cycle pathway [62].

## 5. Conclusions

This study investigated the impact of plateau pika disturbance on the microbial community function and exoenzyme abundance across different alpine grasslands, and it analyzed its role in C cycling pathways. The conclusions can be given as follows:

(1) Microbial community abundance and diversity in alpine grasslands increased as the degradation level increased. Soil pH, sand and C:N are the primary factors regulating bacterial composition. Plateau pika disturbance is conversely related to the effect of sand, soil pH, TN, TC, silt and clay, enhancing the positive effect of NH^4+^-N while reducing the influence of SBD, MBC and NO^3−^-N in regulating bacterial composition. As a result, pika disturbance increases OTUs by 7.5% and 50.5% in LDP (*p* > 0.05) and MDP (*p* < 0.05), while it decreases OTUs by 21.4% in SDP (*p* < 0.05).

(2) Plateau pika disturbance decreases the genes related to chemotrophy and aerobic ammonia oxidation in LDP and MDP, thereby downregulating the exoenzyme abundance-associated simple and complex organic matter decomposition by 9.5% and 13.9% in LDP (*p* > 0.05), and 29.4% and 26.3% in MDP (*p* < 0.05). These changes can partly explain the increase in TC and SOC in these grasslands. Conversely, pika disturbance increases the genes related to aromatic compound degradation in SDP, resulting in the upregulation of exoenzyme abundance related to simple and complex organic matter decomposition by 23.6% and 37.9% (*p* < 0.05), which corresponds to the declines in TC and SOC in SDP.

(3) Plateau pika disturbance can enhance SOC accumulation by upregulating the C cycle pathway of ethanol production in LDP and MDP. However, it upregulates the pathway of pyruvate to CO_2_ conversion in SDP, leading to a negative influence on SOC content. Additionally, pika disturbance also enhances the pathway of methanol reduction to CH_4_ in SDP, suggesting its impact on CH_4_ flux in alpine grasslands.

## Figures and Tables

**Table 1:** Soil physicochemical properties in control and pika-disturbed alpine grasslands.

	UDM	LDC	LDP	MDC	MDP	SDC	SDP
pH	5.39 ± 0.23 ^A^	5.72 ± 0.15 ^A^	5.65 ± 0.07 ^A^	4.81 ± 0.14 ^B^	4.71 ± 0.13 ^B^	7.11 ± 0.17 ^C^	7.21 ± 0.17 ^C^
SWC (%)	160.5 ± 21.6 ^A^	48.4 ± 3.3 ^Ba^	56.1 ± 5.5 ^Bb^	41.6 ± 2.6 ^Ba^	49.3 ± 10.8 ^Bb^	27.9 ± 3.1 ^Ca^	24.5 ± 7.4 ^Cb^
SBD (g/cm^3^)	0.44 ± 0.08 ^A^	1.34 ± 0.16 ^Ba^	1.09 ± 0.12 ^Bb^	1.02 ± 0.04 ^Bb^	0.93 ± 0.03 ^Ca^	1.18 ± 0.12 ^Cb^	1.02 ± 0.04 ^Bb^
Sand (0.05–2 mm%)	34.66 ± 1.67 ^A^	49.76 ± 0.98 ^Ba^	51.23 ± 1.34 ^Ba^	52.45 ± 1.22 ^Ba^	51.89 ± 1.45 ^Ba^	60.89 ± 1.75 ^Ca^	64.56 ± 2.87 ^Ca^
Silt (0.002–0.05 mm%)	47.57 ± 1.09 ^A^	39.32 ± 1.05 ^Ba^	38.08 ± 1.01 ^Ba^	37.05 ± 0.89 ^Ba^	38.76 ± 1.23 ^Ba^	33.23 ± 0.89 ^Ca^	30.45 ± 0.95 ^Ca^
Clay (<0.002 mm%)	18.36 ± 1.22 ^A^	11.21 ± 0.78 ^Ba^	10.15 ± 0.98 ^Ba^	10.24 ± 0.84 ^Ba^	9.86 ± 0.88 ^Ba^	6.54 ± 0.75 ^Ca^	5.89 ± 0.62 ^Ca^
TC (g/kg soil)	202.1 ± 15.3 ^A^	66.83 ± 3.45 ^Ba^	70.15 ± 3.1 ^Bb^	67.38 ± 4.22 ^Ba^	74.19 ± 3.98 ^Bb^	57.36 ± 1.45 ^Ca^	51.45 ± 2.13 ^Cb^
TN (g/kg soil)	8.72 ± 0.15 ^A^	2.41 ± 0.11 ^Ba^	2.83 ± 0.07 ^Bb^	2.43 ± 0.09 ^Ca^	2.68 ± 0.07 ^Cb^	1.81 ± 0.02 ^Da^	2.02 ± 0.02 ^Db^
C:N	23.86 ± 0.45 ^A^	27.55 ± 1.54 ^Ba^	24.56 ± 0.88 ^A^	27.36 ± 1.43 ^Ba^	26.84 ± 0.76 ^Ba^	32.04 ± 0.65 ^Ca^	25.55 ± 0.78 ^A^
SOC (g/kg soil)	129.5 ± 26.1 ^A^	43.69 ± 4.18 ^Ba^	45.55 ± 6.57 ^Ba^	44.92 ± 5.14 ^Ba^	49.64 ± 4.13 ^Bb^	37.05 ± 4.17 ^Ca^	32.19 ± 2.89 ^Cb^
MBC (mg/kg soil)	1295 ± 91.1 ^A^	203.3 ± 17.5 ^Ba^	263.6 ± 23.8 ^Bb^	275.1 ± 27.3 ^Bb^	425.1 ± 40.1 ^Bc^	151.2 ± 14.9 ^Ca^	190.2 ± 16.2 ^Ba^
NH^4+^-N (mg/kg soil)	94.6 ± 11.3 ^A^	35.71 ± 0.98 ^Ba^	52.87 ± 1.12 ^Bb^	66.24 ± 1.67 ^Ca^	74.56 ± 1.88 ^Cb^	69.76 ± 2.45 ^Ca^	62.64 ± 1.54 ^Ca^
NO^3−^-N (mg/kg soil)	341.3± 19.7 ^A^	53.89 ± 1.09 ^Ba^	64.65 ± 1.65 ^Bb^	42.34 ± 1.01 ^Ca^	48.12 ± 0.98 ^Cb^	20.85 ± 0.65 ^Da^	39.56 ± 0.98 ^Db^

Note: UDM, undegraded alpine grassland; LDC, MDC and SDC, control sites of lightly, moderately and severely degraded grassland; LDP, MDP and SDP, pika-disturbed sites of lightly, moderately and severely degraded grassland. SWC, soil water content; SBD, soil bulk density; TC, total C content; TN, total nitrogen content; C:N, ratio between TC and TN; SOC, soil organic carbon; MBC, microbial biomass C; NH^4+^-N, ammonium nitrogen; NO^3−^-N, nitrate nitrogen. ANOVA with two-sided and post hoc tests was performed to determine the significant differences among groups. The uppercase letters indicate the significant difference among varied degraded alpine grasslands; the lowercase letters refer to the significant differences between control and plateau pika disturbed alpine grasslands (*p* < 0.05).

**Table 2:** Bacterial abundance and diversity across different alpine grasslands.

Alpine Grassland	OTU	*α* Diversity			*β* Diversity		
		Chao_1	Faith_pd	Shannon_ Winner	Bray_curtis	Weighted Unifrac	Unweighted Unifrac
UDM	4724 ± 213 ^A^	6453 ± 200	214.6 ± 5.5	10.36 ± 0.23	0.811 ± 0.016	0.104 ± 0.00	0.534 ± 0.008
LDC	4590 ± 28 ^A^	5883 ± 87	195.8 ± 2.6	10.60 ± 0.02	0.733 ± 0.009	0.093 ± 0.004	0.526 ± 0.001
LDP	4934 ± 294 ^A^	6129 ± 435	201.2 ± 30.7	10.43 ± 0.61	0.759 ± 0.032	0.108 ± 0.018	0.542 ± 0.038
MDC	3745 ± 246 ^Ba^	6294 ± 345	202.7 ± 18.8	10.83 ± 0.29	0.733 ± 0.001	0.100 ± 0.006	0.516 ± 0.001
MDP	5637 ± 285 ^Bb^	6623 ± 488	216.2 ± 24.5	10.97 ± 0.22	0.735 ± 0.023	0.097 ± 0.010	0.516 ± 0.010
SDC	5831 ± 104 ^Bb^	6694 ± 501	221.2 ± 17.3	10.69 ± 0.17	0.744 ± 0.010	0.102 ± 0.001	0.526 ± 0.013
SDP	4583 ± 161 ^A^	6056 ± 173	201.0 ± 2.2	10.39 ± 0.19	0.817 ± 0.016	0.114 ± 0.009	0.538 ± 0.004

Note: The uppercase letters indicate the significant difference among varied degraded alpine grasslands; the lowercase letters refer to the significant differences between control and plateau pika disturbed alpine grasslands (*p* < 0.05).

## Data Availability

All the data used in this study are available from the corresponding author on reasonable request.
